# Prevalence of intestinal parasite among patients attending two hospitals in French Guiana: A 6-year retrospective study

**DOI:** 10.1371/journal.pntd.0009087

**Published:** 2021-02-05

**Authors:** Alolia Aboikoni, Manon Allaire, Dominique Louvel, Denis Blanchet, Thong Dao, Jean-François Carod, Magalie Demar

**Affiliations:** 1 Service d’Hépato-gastroentérologie et de nutrition, CHU Côte de Nacre, Caen-France; 2 Centre de Recherche sur l’Inflammation,UMR 1149, INSERM, Université Paris-Diderot, ERL CNRS 8252, Paris-France; 3 Service de d’Hépato-gastroentérologie, Centre Hospitalier de Cayenne, Cayenne, Guyane française; 4 Laboratoire Hospitalo-Universitaire de Parasitologie Mycologie, Centre Hospitalier de Cayenne, Cayenne, Guyane française; 5 Service de biologie, CH Ouest Guyanais, Saint Laurent du Maroni, Guyane française; 6 Ecosystème amazonien et pathologie tropicale, EA 3593, EPaT, Université de Guyane, Cayenne, Guyane française; Istituto Superiore di Sanità, ITALY

## Abstract

**Introduction:**

Intestinal parasitic diseases are a global health problem. Due to its equatorial climate, vast territory with isolated areas and the precariousness of its population, intestinal parasitosis is considered to be a major issue in French Guiana but only few data are available and these mainly focus on specific population. We aimed at determining the parasitic index and at describing the characteristics of these infections in order to develop preventive strategies.

**Material and methods:**

We retrospectively analysed all the parasitological samples recorded in the register of the two main laboratories of French Guiana between 2011 and 2016. The parasitic index was the percentage of parasitised patients in comparison with the total number of subjects studied. A patient who underwent several positive parasitological examinations was considered only once in the analysis at the time of the first sampling.

**Results:**

A total of 15,220 parasitological samples of 9,555 patients were analysed and 2,916 were positive in 1,521 patients. The average infestation rate and parasitic index were 19.2% and 16.0%, respectively. The parasitic index remained stable between 2011 (18.2%) and 2016 (18.3%). The patients were mainly men (66.4%), with a median age of 33.0 years (26.3% of patients were under 18 years of age) and lived mainly in the Central Agglomeration (48.2%) and in West Guiana (37.4%). Hookworms were the most common parasite (25.2%) followed by *Entamoeba coli* (13.3%), *Strongyloides stercoralis* (10.9%) and *Giardia intestinalis* (10.8%). Among the infected patients, 31.0% presented mixed infections and 67.5% of them had at least one pathogenic parasite. The patients aged from 0 to 18 years presented significantly more polyparasitism (30.9%) than monoparasitism (24.3%, p<0.001). *Ancylostoma sp* and *Strongyloides stercoralis* were mainly diagnosed during the rainy season (59.5% and 64.7% respectively), in men (78.6% and 81.1% respectively) and in patients aged from 18 to 65 years (86.6% and 76.6% respectively) whereas, *Giardia intestinalis* infected mostly children under 5 years (59.5%) of age.

**Conclusion:**

Although it may not be representative of the entire Guyanese population, the parasitic index remained high and stable from 2011 and 2016 and it justifies the need for an active prevention program as it was already done in the other French overseas departments such as Martinique and Guadeloupe.

## Author summary

Intestinal parasitic diseases are a global public health problem which mostly affect developing countries where adequate water and sanitation facilities are lacking. Due to its equatorial climate, vast territory with isolated areas and the precariousness of its population, intestinal parasitosis is considered to be a major issue in French Guiana and came to be frequently detected during hospitalisations. However, only few data are available and no integrated control programs have been conducted.

This is the first study that has been exploring and reporting epidemiological data on intestinal parasitic infections in French Guiana covering much of the territory. This work reports an average parasitic index of 16.0%, which was stable between 2011 (18.2%) and 2016 (18.3%) with a majority of *Ancylostoma sp*, *Strongyloides stercoralis* and *Giardia intestinalis*. Although it may not be representative of the entire Guyanese population, the parasitic index remained high and stable from 2011 to 2016 and it justifies the need for an active prevention program as it was already done in the other French overseas departments such as Martinique and Guadeloupe.

## Introduction

Intestinal parasitic diseases are a global public health problem caused by intestinal helminths and protozoan parasites which mostly affect developing countries where adequate water and sanitation facilities are lacking. If mortality from these infections remains low, morbidity will be significant and will depend on the severity of the infection and the associated clinical symptoms. In fact, digestive disorders such as, diarrheas and abdominal pains can be observed along with severe anaemia or malnutrition in case of a prolonged infection. In addition, such infections, when they are severe, can also impact the psychomotor development of children [1–3].

French Guiana is an Amazonian region located in South America, separated from Brazil by the Oyapock River from the eastern side, and from Surinam by the Maroni River from the western side (S1 Fig). In 2016, its population had been estimated to 269,352 of whom, 61.1% lived in the Central Agglomeration near the Atlantic coast and 34.5% along the Maroni River [4]. Due to its equatorial climate, vast territory with isolated areas and the precariousness of its population, intestinal parasitosis constitutes a real issue in French Guiana and can cause high morbidity especially inside the children population as described in the literature [5–7]. However, very few studies focusing on this issue are available, and they were about specific populations such as the Amerindian people [8] and the militaries [9], pointing that no public measures have been conducted in this region in order to contain the spreading of intestinal parasites. To date, no updated surveys cover the intestinal parasitic infections in the entire region of French Guiana or give a global view of this public health issue.

In the 1980s, an integrated control program associating sanitary education, detection and treatment of the patients with intestinal parasitosis was conducted in Martinique and Guadeloupe, two French overseas departments in the Caribbean area. This program led to a strong reduction of the prevalence of parasitic infections [10,11]. Therefore, such similar strategy could be developed in French Guiana regarding the current epidemiological situation.

Thereby, the objectives of our study were (i) to offer an updated report on the intestinal parasitic infections using the data records of the two main hospitals of French Guiana, (ii) to determine the parasitic index (PI) through the whole territory of French Guiana from 2011 to 2016 in order to develop preventive strategic lines.

## Materials and methods

### Ethics statement

We did not seek approval for Institutional review board because the retrospective analysis of anonymised data is allowed by French regulation and all patients were informed of their right to refuse that their results may be used for subsequent research. Posters in local languages and cartoons were used to convey this information in the wards and at the laboratory. The data analysed for this study were anonymised.

### Territory distribution

This study covered the entire territory of French Guiana, a French department situated in the Amazon basin in the Guiana Shield. According to parameters such as biotopes, geographical specificities (access by planes, pirogues…), access of care (number of medical facilities) and the socio-cultural characteristics of the populations, we defined five different geographical areas: the East Guiana, the Central Agglomeration, The Savannah region, the West Guiana and the Central region. All their characteristics are summarised in the S1 Table.

### Description of the health system

In French Guiana, there are 3 general hospitals mainly in the littoral (the Centre Hospitalier de Cayenne (CHC), the Centre Hospitalier de Kourou [CHK] which has replaced the private hospital of Kourou since January 2018 and the Centre Hospitalier de l’Ouest Guyanais [CHOG] in Saint Laurent du Maroni). There are also 17 health centers (S1 Fig). In the inland of French Guiana, there are no laboratories and most of the tests including the parasitological examination of feces are sent to the two main hospitals laboratories of Cayenne and Saint-Laurent-Du-Maroni at 8 degree Celsius within 24–48 hours. However, it is important to point out that transferring the samples can affect the diagnostic of amoebiasis due to the delayed investigation of the fresh stool samples. Only point-of-care testing for biochemistry and haematology are practiced in the two main health centers, Maripasoula and St Georges. The 5 private laboratories also conduct these analyses and take care of less than 10% of the total of parasitological analyses. Unlike in Martinique and Guadeloupe, neither public measure nor health policies for the control of intestinal parasites such as mass drug administration were experienced in French Guiana.

### Patients’ selection and data collection

We retrospectively analysed the whole of the parasitological examinations recorded in the register of the two main hospital laboratories between January 1, 2011, and December 31, 2016. According to the hospital-based protocol, the parasitological examinations were conducted on patients because of symptoms evoking parasitic infections such as diarrheas, abdominal pain, severe anaemia, or when there was a notification of hypereosinophilia in asymptomatic patients, mostly of fortuitous finding. All the stool and digestive fluids samples of laboratories based-database were included. The data of each patient such as their gender, their date of birth and their place of living, as well as the date of the analysis and the type of carried out parasitological examination were recorded. In this record, the clinical data, the treatments provided and the evolution of patients’ health were not registered at the time when the parasitological analysis was conducted and we were not able to provide such data due to anonymisation of the data and the retrospective design of the study. The patients with mixed infections were defined by the coexistence of two or more parasites coexisting in the same sample or in several successive samplings in the week following the first sampling. The data from the rainy season concerned samples collected between December and June and those from the dry season were collected between July and November.

### Parasitological techniques or analyses

#### Parasitological examinations of the stool samples

According to the laboratory protocol, the macroscopic examination of fresh stool specimens (color, consistence, presence of blood, mucus or adult parasites) was done. Then, the specimens were examined through a microscope analyzing: i) direct smear, ii) the Bailanger’s concentration for protozoa and helminths research, iii) the Baermann’s technique for *Strongyloides stercoralis*, and iv) the kato concentration for helminth eggs. The search for *Cryptosporidia* was systematic using a modified Ziehl-Neelsen stain. In case of a suspicion of amoebosis, a direct examination within 2 hours or the use of MIF coloration was performed and the sample kept at +4°C when the reading of the exam was postponed.

#### Examination of digestive fluids

The examined digestive fluids were mainly duodenal fluid collected during an upper digestive endoscopy. The suction of duodenal fluid was mainly carried out when there was an aspect of duodenitis. They were then freshly examined on microscope directly and after a centrifugation process. In addition to the ova and larvae, we included in this group the adult parasites which had been seen macroscopically during an endoscopy.

### Statistical analyses

Some parameters such as the infestation rate (IR) and PI were calculated. The IR corresponded to the percentage of positive samples compared to the total number of samples analysed. The IR gives an idea of the proportion of positive samples. However, most parasitology textbooks and laboratory manuals recommend the examination of at least three independently collected stool specimens to maximise the sensitivity of detecting intestinal parasites [12] especially when multiple specimens were examined. In the present study, patients often presented several stool samples several days in a row. Consequently, in order to avoid an overestimation of our results, we calculated the PI which corresponded to the percentage of parasitised patients in comparison with the total number of subjects studied. Thus, a patient with several positive parasitological examinations was evaluated only once in the analysis at the time of the first sampling. As fecal examinations were performed when there where fortuitous findings of hypereosinophilia in asymptomatic patients or in symptomatic patients with or without hypereosinophilia, the IR and PI did not estimate the prevalence of intestinal parasites in the whole population of French Guiana but in this specific population of patients.

The characteristics of the patients were presented as means ± standard deviation (SD) or medians [interquartile range (IQR)] for continuous variables, and as numbers (percentages) for categorical data. The categorical variables were compared using the χ^2^ test or Fisher’s exact test if necessary. All statistical analyses were performed using Stata 13.0 (StataCorp, College Station, TX). A *P* value <0.05 was considered to be statistically significant.

## Results

### Characteristics of parasitological samples

**Fig 1:**
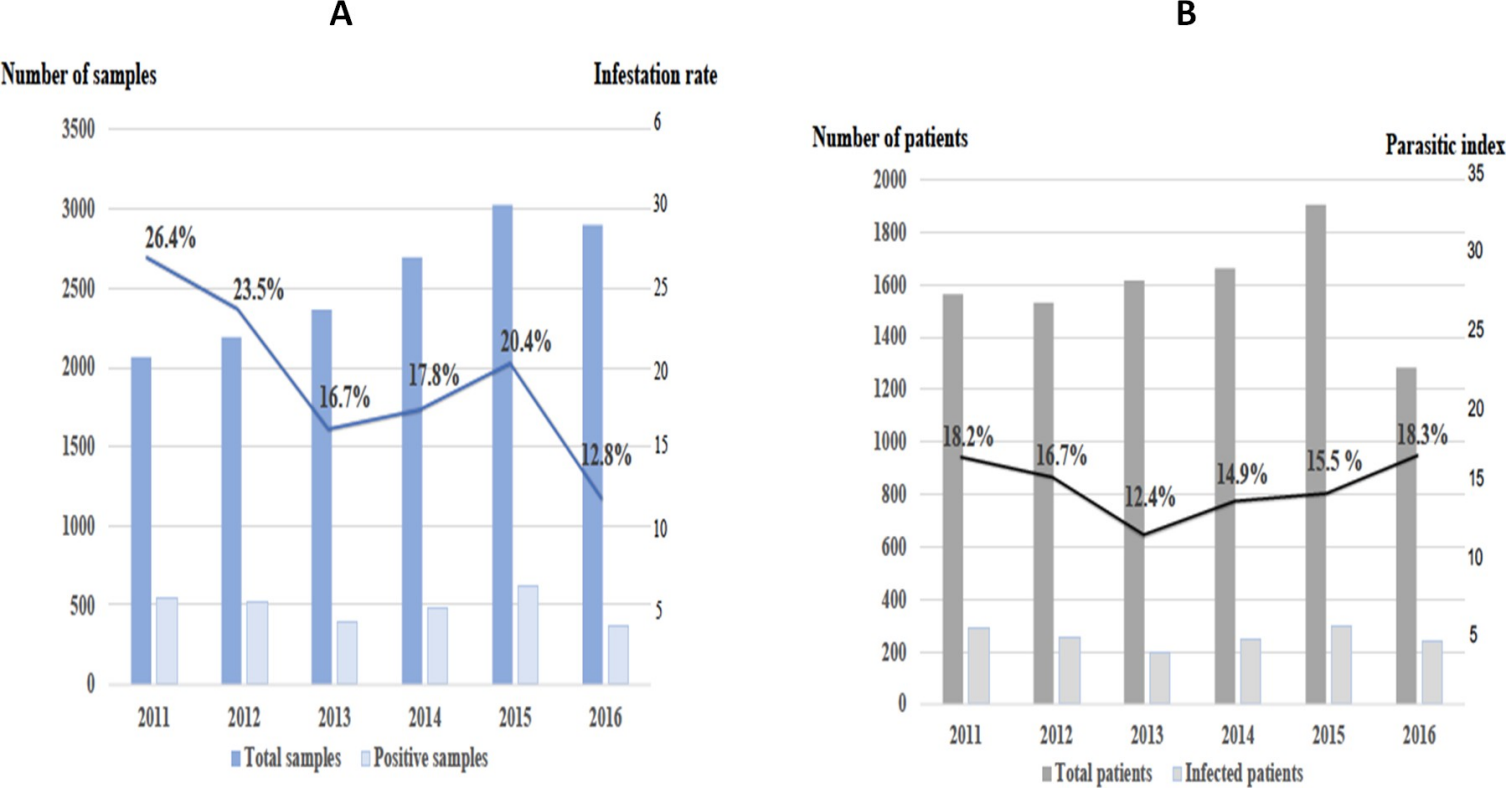
Evolution of the infestation rate (A) and the parasitic index (B) between 2011 and 2016.

We studied retrospectively over the period of January 1, 2011 to December 31, 2016, 15,220 parasitological samples of 9,555 patients from the CHC and CHOG laboratory registries. The majority of them was analysed from the CHAR laboratory data (77.4%). The increase in the number of samples collected was linear until 2015 and was followed by a slight decrease until 2016. Among these 15,220 samples, 2,919 were positive for 1,521 patients. The average infestation rate was 19.2% and a decrease was observed between 2011 (26.4%) and 2016 (12.8%) (Fig 1A).

The samples were essentially stool samples but 2,075 specimens were digestive fluids collected during an upper endoscopy as duodenal fluid or a colonoscopy. It was positive for 169 patients with the identification of the adult parasite in 41 patients and ova/larvae in 128 patients. Among them, 67 patients were positive for *Ancylostoma* (39.6%), 56 for *Strongyloides stercoralis* (33.1%), 6 for both *Ancylostoma* and *Strongyloides stercoralis* (9.0%) and 20 for *Ascaris lumbricoides* (11.8%), 20 for others parasites (*Trichurius trichura*, *Enterobius vermicularis*, *Giardia intestinalis*, *Entamoeba coli*, *Entamoeba hartmanni*, *Entamoeba histolytica/dispar)*. Among the positive patients who underwent an endoscopy examination, 45 showed positive stool samples from the same parasites.

### Characteristics of infected patients

An infected patient was evaluated only once when he presented multiple positive samples and the characteristics of the 1,521 parasitised patients who were subjected to fecal or endoscopic examinations are detailed in Table 1. Several successive samples were positive for the same parasite within 570 patients. The diagnosis of intestinal parasitosis was more common among men (66.4%). The average age at diagnosis was 33 years [2 months-94 years] and 24.6% of the patients were under 18 years of age. The number of positive diagnosis was higher during the rainy season. The parasitised patients lived mainly in the Central Agglomeration (48.2%) and in West Guiana (37.4%). The average parasitic index was of 16.0%. A decrease was observed between the years 2011 (18.2%) and 2013 (12.4%), before an ascent until 2016 (18.3%) (Fig 1B).

**Table 1:** Characteristics of infected patients.

	Available data	Number of patients n = 1,521
**Male gender** (n, %)	1,498	994 (66.4)
**Median age** (years) (n, %)	1,519	33 [2 months- 94 years]
< 1 years		50 (3.3)
1–5 years		215 (14.2)
6–17 years		133 (8.8)
18–64 years		1.030 (67.7)
> = 65 years		91 (6.0)
**Years of diagnosis** (n, %)	1,521	
2011		286 (18.8)
2012		255 (16.8)
2013		201 (13.2)
2014		248 (16.2)
2015		296 (19.5)
2016		235 (15.5)
**Rainy Season** (n, %)	1,521	917 (60.3)
**Living areas** (n, %)	1,484	
Central Agglomeration		716 (48.2)
West Guiana		555 (37.4)
East Guiana		175 (11.8)
Central region		8 (0.5)
Savannah region		7 (0.5)
Others*		23 (1.6)

* France Metropolis, Surinam, Brazil, French West Indies and Africa.

### Characteristics of the parasites observed

**Fig 2:**
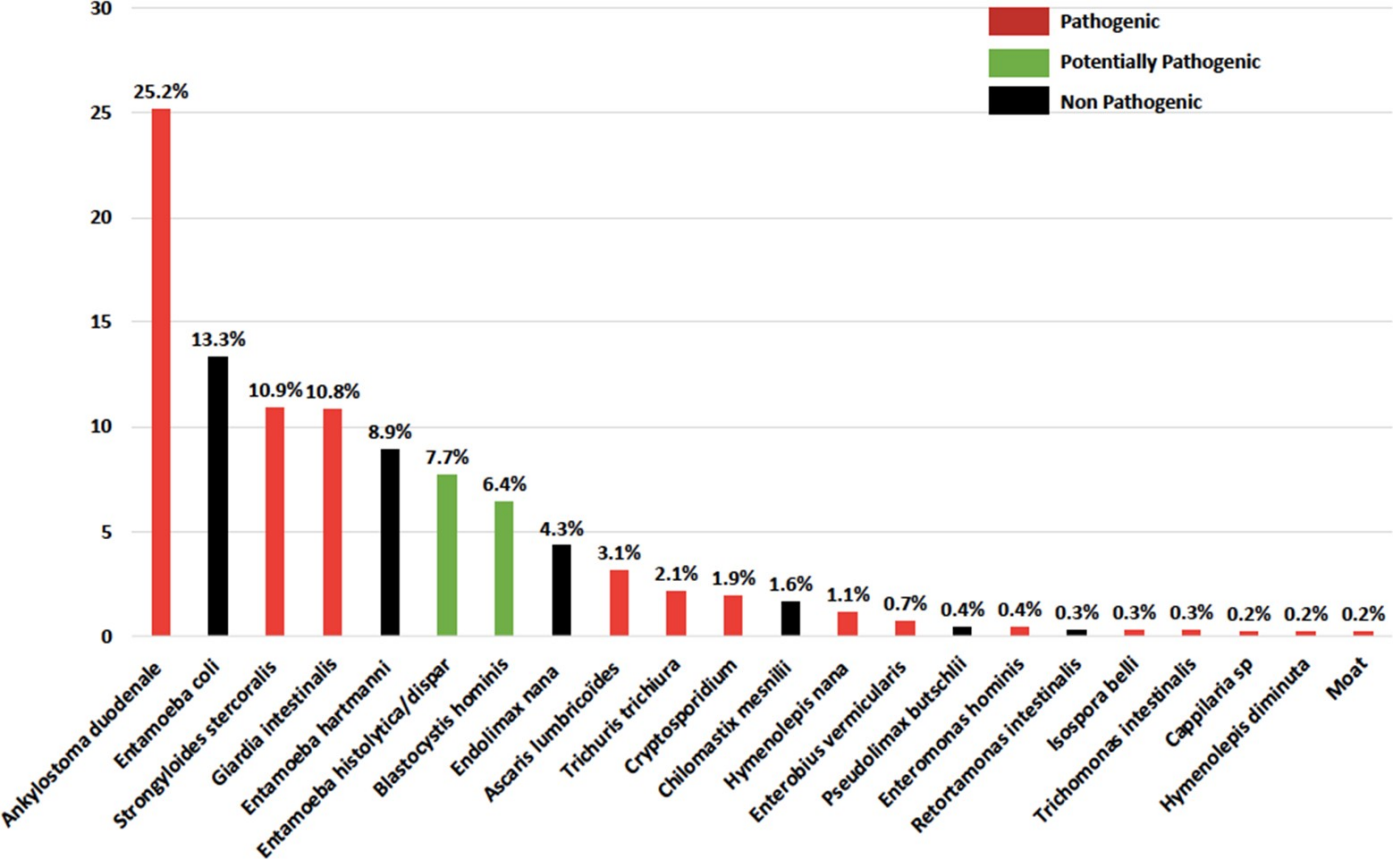
Type of parasites observed.

**Fig 3:**
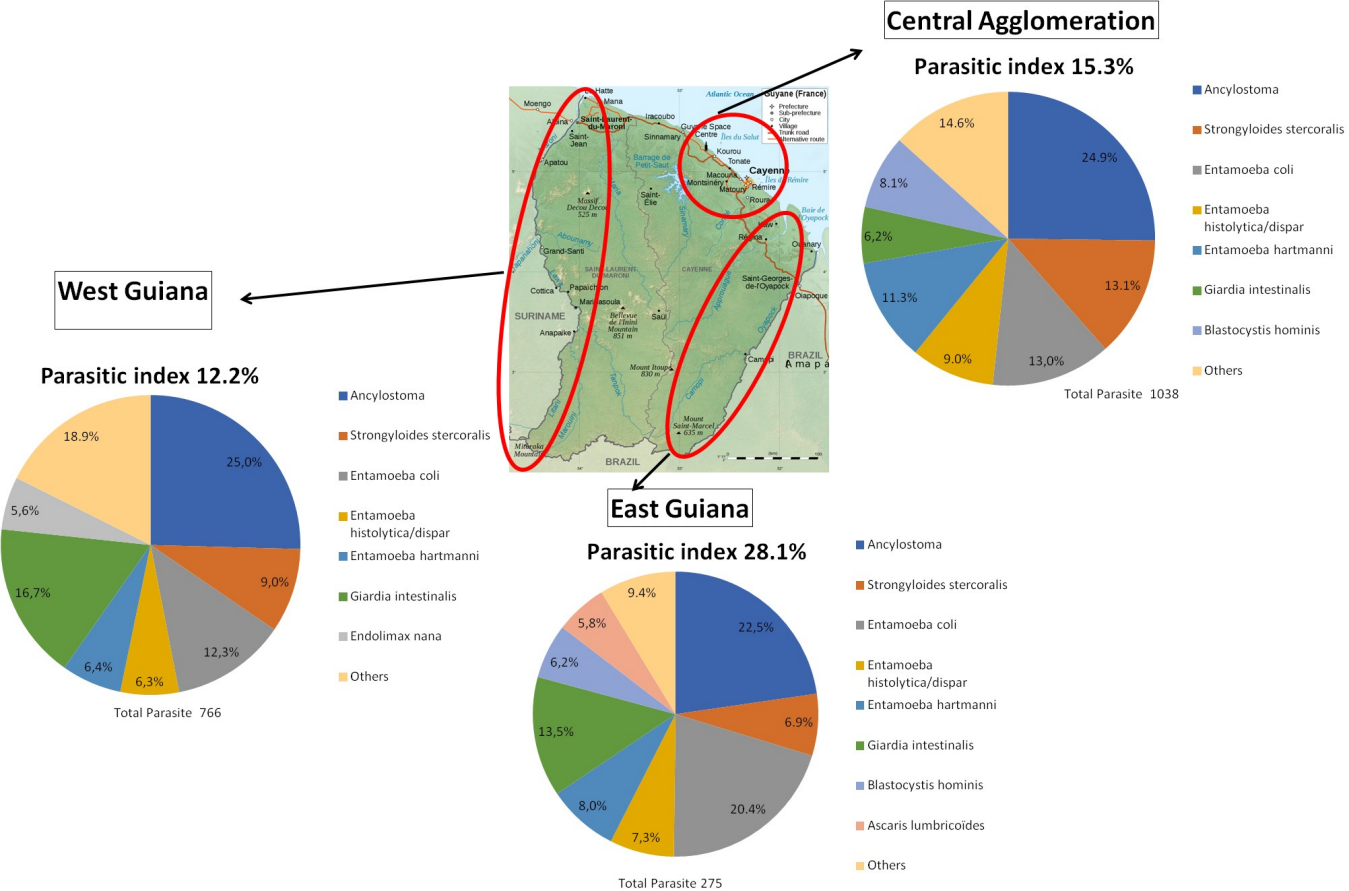
Distribution of parasites and parasitic index according to the 3 main geographical areas.

We observed 2,166 parasites among the 1,521 parasitised patients. This data correspond to the number of parasites found in positive samples and not to the number of species. Among the parasitised patients, 471 (31.0%) had mixed infections. The identified parasites are shown in Fig 2; 56.5% were protozoa (23.5% were pathogenic) and 43.5% helminths (100% were pathogenic). Hookworms were the most common (25.2%) followed by *Entamoeba coli* (13.3%), *Strongyloides stercoralis* (10.9%) and *Giardia intestinalis* (10.8%). The characteristics of each parasite according to the gender, age, seasons and areas are presented in S2 Table. The distribution of parasites was different according to the geographical areas: high ecological diversity in the East (PI 28.1%), West (PI 12.2%) and Central Agglomeration (PI 15.3%) as exposed in Fig 3. For Savannah region and the Center region, the PI were 28.0% and 33.3% respectively but few patients and few parasites were recorded. Seven parasites among seven patients were diagnosed in the Savannah region (85.7% of *Strongyloides stercoralis* and 14.3% of *Ancylostoma*) and nine parasites among seven patients in the Center region (77.8% of *Ancylostoma*, 11.1% of *Strongyloides stercoralis* and 11.1% of *Entamoeba hartmanni*).

### Characteristics of patients with mixed infections

Among the infected patients, 471 (31.0%) patients were infected with 2 or more parasites. Among them, 317 patients had 2 parasites (67.3%), 114 had 3 parasites (24.2%), 29 had 4 parasites (6.2%), 9 had 5 parasites (1.9%) and 2 had 6 parasites (0,4%). The 5 most frequent combinations were *Ancylostoma* and *Giardia intestinalis* (8.7%), *Ancylostoma and Strongyloides stercoralis* (7.2%), *Ancylostoma and Entamoeba coli* (5.3%), *Entamoeba coli and Entamoeba hartmanni* (4.2%), *Entamoeba coli and Entamoeba histolytica/dispar* (3.4%) (S3 Table).

The patients aged from 0 to 18 years have presented significantly more polyparasitism (30.9%) than monoparasitism (24.3%, p<0.001). Polyparasitism was significantly much more common in Eastern Guiana (14.9% versus 8.7%, p<0.001) whereas it proved less common in Central Agglomeration (44.8% versus 51.4%, p = 0.02) (S4 Table).

### Characteristics of the 3 most common pathogenic parasites

#### Ancylostomidae

Among the parasitised patients, 546 of them have presented positive samples for *Ancylostoma sp*. Most of the diagnoses were made among men (78.6%), in patients aged from 18 to 65 years (86.6%). The Specific parasitic index was higher in Central Agglomeration and in West Guiana. We have observed a peak frequency in 2015 before a decrease in the number of patients was reached until 2016. It was the most common parasite observed in each region except the Savannah area (Fig 3).

#### Strongyloides stercoralis

Two hundred and thirty-five patients were infected with *Strongyloides stercoralis*. The majority of cases was composed of men (81.1%) and patients aged from 18 to 65 years (76.6%) in the rainy season (64.7%). The specific parasitic indexes for *Strongyloides stercoralis* were higher in the Savannah region and Center region. It was the first pathogenic parasite in the Savannah region, the second in the Central Agglomeration and the third in West and East Guiana (Fig 3).

#### Giardia intestinalis

Two hundred and thirty-three patients were parasitised by *Giardia intestinalis*. The patients were predominantly male (61.1%) and children under 5 years (50.9%) in the rainy season (64.8%). The presence of the parasite was most common in the areas near the river (West and East Guiana) (Fig 3).

## Discussion

This is the first study that shows an epidemiological report on the intestinal parasitic infections using the hospital data records in the entire French Guiana. This retrospective study which covered the entirety of the region, reported an average PI of 16.0% in patients attending the two main hospitals of French Guiana and for whom there was an indication of intestinal parasitological investigation. The PI was considered stable between 2011 (18.2%) and 2016 (18.3%). The most common pathogenic intestinal parasites were *Ancylostoma sp*, *Strongyloides stercoralis* and *Giardia intestinalis*. A third of the patients have presented mixed infections and among them a third was under 18 years of age. The morbidity of these parasites is well known [2,3,5–7]. Therefore, these data raise the need for the development of preventive measures in this French department that presents a real heterogeneity and reserved socioeconomical status [13].

One of the strengths of the study was the methodology chosen for the interpretation of data leading to an average PI of 16.0%. In most of the studies, the statistical methodology was not well detailed, and these studies took into account stool samples to estimate the prevalence of intestinal parasitosis [10,11,14]. However, as a patient might have showed several successive positive samples of the same parasite, this might have generated higher values as we observed in our study with an IR higher than the PI. In order to have a better appreciation of the intestinal parasitosis infection, we took into account the number of patients and calculated the PI. Indeed, we chose to evaluate a patient only once and regardless of the number of positive samples for each PI calculation, that is to say that a patient with several positive samples was counted once and was considered polyparasitic when different parasites were found within the samples. Very few studies [15–17], just as the one we conducted, took into account the number of parasitised patients out of the total number of patients studied over a target period. Compared with data from the literature using the same strategy for PI calculation, in the South American continent, despite its distance from the Amazon and French Guiana, the PI in Rio Do Janeiro (Brazil) was close to our results (17.5%) [16]. However, for a locality with a low socio-economical level in the region of Belem, a Brazilian metropolis close to French Guiana, Cardoso *et al*. reported in 2015 a PI of 64.7% [15]. In this study, patient with tuberculosis and their neighbours gave a unique stool sample upon which the analysis was conducted and the authors have shown the link between a low socioeconomical level and pathologies such as tuberculosis and intestinal parasitosis. Similarly, the PI was 53.6% in Suriname and 43.5% in Guyana in a small study concerning children [18,19].

In the other French overseas departments, the infestation rates were of 6.7% in Guadeloupe from 1991 to 2003 and of 8.7% in Martinique from 1994 to 1995, a lower rate than the one observed in our study [10,11]. These significant differences could be first explained by a preventive program developed in the 1980s in the two Caribbean departments and also by the presence of a climate more likely to lead to parasitosis as well as more precarious sanitary and social conditions in French Guiana.

In Carme *et al’s study*, published in 2002, 92% of the 138 Wayampi Indians studied from French Guiana had intestinal parasites. The most common ones were *Entamoeba coli* (58.0%), *Ancylostoma sp* (57.1%) and *Strongloides stercoralis* (16.3%) [8]. In our study, conducted approximately 15 years later, the region East Guiana also encompassed this area and we found out that the rate of every parasite was lower. This consequence is probably due to the fact we included St Georges which is much more urbanised and economically developed. Moreover, the Amerindian population studied by Carme et al. really lived in an isolated area with a very close contact with the Amazonian forest, and these people were walking barefoot. *Entamoeba coli* and *Ancylostoma sp* remained the 2 most frequent parasites. The frequency of *Giardia intestinalis* was similar (14.5% vs 13.6%) in relationship to the consumption of contaminated water and the proximity of the river. The frequency of *Hymenolepis nana* was higher in the Carme’s study probably because of the large proportion of children presented in the study.

Interestingly, we have observed a high diversity in the parasitological ecology for the Central Agglomeration, East and West Guiana, unlike the Center and Savannah region. This fact can be related to the small amount of patients coming from these last regions in our study.

In our study, *Giardia intestinalis* was the second pathogenic parasite diagnosed in the Eastern and Western parts of the French Guiana. This observation could result from the large number of rivers in these areas. Indeed, the route of transmission of this parasite is the ingestion of contaminated water and food. In these regions, people commonly consume the water from rivers which is polluted by everyday life without resorting to preventive measures, particularly because of the lack of sanitary facilities. In our study, the target population affected by *Giardia intestinalis* was children under 5 years-old, living in Eastern and Western Guiana in the rainy season. It is known that *Giardia intestinalis* is more prevalent in children and a contact with children could also promotes the parasitic transmission [20]. Some preventive measures could be implemented and these may include informing the parents, raising the awareness of consulting in centers of maternal and child protection, school children learning about hygiene measures in pleasant ways (game, role plays).

Hookworms were the most common parasites in all the geographical areas except for the Savannah areas where anguillulosis was dominant. A lower rate of hookworm could have been expected in the Central Agglomeration, which is an urban area compared to other regions, given the mode of transcutaneous transmission. A potential explanation could be the large population’s migrations in recent years in French Guiana. In fact, the Central Agglomeration, the most developed area of the department, welcomed the populations of the inside but also the immigrants of the bordering countries, people that could have been in contact with hookworms before their migration.

Regarding the anguillulosis, the literature gives us rather disparate infectious rates going from 0.45% to 59.4% [10,14,15,17,18,21,22]. This disparity could be first explained by the methodology chosen for the analysis (calculation of IR versus PI) and by the difference in the diagnostic methods used. In fact, the current standard Baerman Gold technique was not systematically performed in some of the studies. Moreover, the high rate observed in our study could be explained partly by the difficulty in eradicating the parasite because of its internal parasitic cycle and secondly by the high prevalence of HTLV-1 infection in French Guiana, which is a risk factor of persistence of anguillulosis accounting for an increase in morbidity, mortality and development of cancer [23].

Because of the significant morbidity of these parasitic diseases, our series underlines the need of a proper survey in French Guiana, including clinical data, dynamics of contamination, and the evaluation of the intensity of the infection with the analysis of the eggs per gram of feces, and anaemia level. Indeed, this should better target the preventive measures to be provided such as providing information about the mode of contamination of the parasite, and the proscription of walking barefoot, and could then be implemented as in Guadeloupe and Martinique where the measures implemented allowed a reduction of their prevalence [10,11,24].

However, this study presents some limitations. First, only parasitological data from the CHC and CHOG laboratories were analysed. Yet, these two structures are the reference laboratories for parasitology in French Guiana and receive samples from all over the country as evidenced by the identified residential areas of the patients. Only 5 private laboratories carry out parasitological analyses and represent less than 10% of the global sample. Secondly, in the isolated regions, because of the distance between the local health center and the more developed health structures, it is common to prescribe an antiparasitic treatment in the presence of digestive symptoms without parasitological examination, thus generating a possible underestimation of our data. Thirdly, we only considered patients who were subjected to faecal and endoscopic examination and not the whole population of French Guiana. Finally, we could not access the symptoms, treatment and follow-up of the patients and we were not able to establish data on re-infestation, morbidity and mortality related to intestinal parasitosis. Indeed, a larger sample with relevant medical information is needed to have a better view of the public health issue. Several criteria such as the clinical presentation, the psychomotor impact, the correlation with the geographical parasite’s distribution and the response to treatment could be analysed.

**To sum up**, this work on intestinal parasitosis in French Guiana shows the potential burden that could represent the intestinal parasitic infection in this French department with a high but stable PI from 2011 to 2016. Further studies with detailed description of the infection may explain more accurately the prevalence and the morbidity of the disease in French Guiana. An active prevention program such as the one which was already in the other French overseas departments should be implemented.

## Supporting information

S1 Fig Health structures in French Guiana. (TIF)

S1 Table Geographical areas. (DOCX)

S2 Table Characteristics of parasites. (DOCX)

S3 Table The most common combinations of parasites. (DOCX)

S4 Table Characteristics of patients with mixed infections. (DOCX)
