# Supplementary figures and images for: Prevalence of intestinal parasite among patients attending two hospitals in French Guiana: A 6-year retrospective study
Source: PLoS Negl Trop Dis. 2021 Feb 5;15(2):e0009087. doi: 10.1371/journal.pntd.0009087 (PMC7891781; doi:10.1371/journal.pntd.0009087)

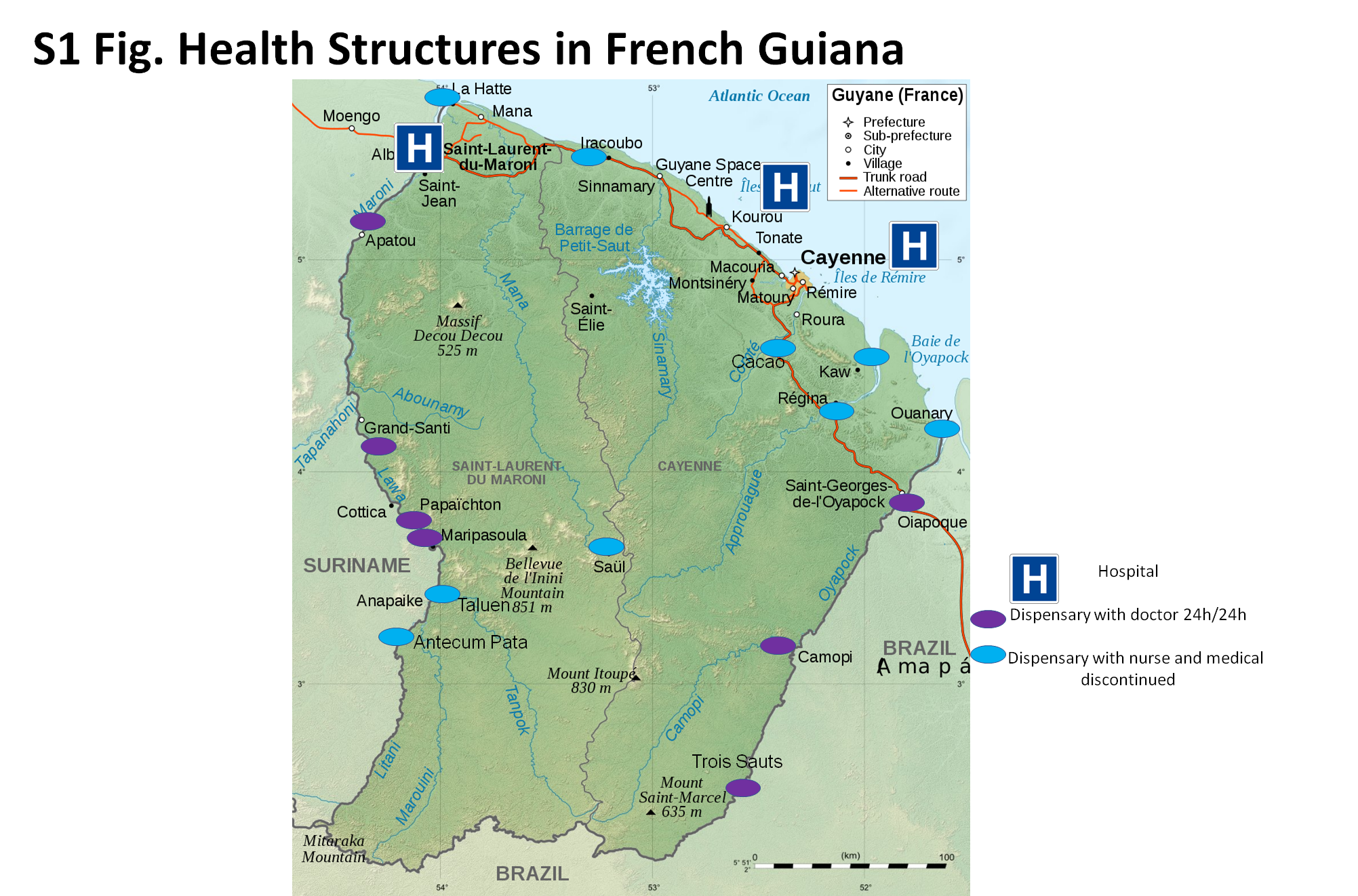

Supplement: S1 Fig — (TIF) [file pntd.0009087.s001.tif]
