# Supplementary material for: Prevalence of intestinal parasite among patients attending two hospitals in French Guiana: A 6-year retrospective study
Source: PLoS Negl Trop Dis. 2021 Feb 5;15(2):e0009087. doi: 10.1371/journal.pntd.0009087 (PMC7891781; doi:10.1371/journal.pntd.0009087)
Supplement: S1 Table — (DOCX) [file pntd.0009087.s002.docx]

**S1 Table. Geographical areas**

|  | **East Guiana** | **Central agglomeration** | **Savannah region** | **West Guiana** | **Center region** |
| --- | --- | --- | --- | --- | --- |
| **Location** | Brazilian frontier, along the Oyapock River. | On the littoral and its surroundings | On the littoral | Surinamese frontier, along the Maroni River with the two areas: upper and lower Maroni | Inlands areas with difficult accessibility |
| **Biotope** | Rural and forestrian | Urban et rural | Rural | Rural, forestrian, urban | Forestrian |
| **Geographical specificities** | Accessible by road and canoe. Border with Brazil: problem of immigration. Some communes are isolated and accessible only by canoe. Lack of sanitation and access to drinking water for some areas | Most developed and populated area of the department. | Two Small rural communes with few inhabitants.  Accessible by road. | Accessible by road and canoe. Border with Surinam : problem of immigration.  Lack of sanitation and access to drinking water for some areas. | Inlands areas |
| **Type of populations** | Amerindian,  Brazilian, Creole | Multi ethnic | Creole | Marrons : African slaves descendants, Amerindian, Hmong, immigrants | Creole |
| **Access to care** | 4 dispensaries | 1 public hospital  3 private hospital | 1 dispensary | 1 public hospital in Saint Laurent du Maroni ; most of the communes are far from this hospital  and have only dispensary | 1 dispensary |
| **Number of inhabitants in 2016**[4] | 6,956 | 164,486 | 4,765 | 92,995 | 298 |
| **Main cities** | Camopi, Ouanary,  Regina-Kaw, Saint Georges, Trois-sauts | Cacao, Cayenne, Kourou, Macouria, Matoury, Montsinerry, Remire Montjoly, Roura, | Sinnamary,  Iracoubo | Apatou, Grand Santi, Mana  Maripasoula, Papaichton, Saint Laurent du Maroni, | Saint-Elie, Saul |
