# Supplementary material for: Prevalence of intestinal parasite among patients attending two hospitals in French Guiana: A 6-year retrospective study
Source: PLoS Negl Trop Dis. 2021 Feb 5;15(2):e0009087. doi: 10.1371/journal.pntd.0009087 (PMC7891781; doi:10.1371/journal.pntd.0009087)
Supplement: S2 Table — (DOCX) [file pntd.0009087.s003.docx]

**S2 Table. Characteristics of parasites**

|  |  | **Sex gender** | | | **Age** | | | | | | **Season** | | **Geographical distribution** | | | | | | |
| --- | --- | --- | --- | --- | --- | --- | --- | --- | --- | --- | --- | --- | --- | --- | --- | --- | --- | --- | --- |
|  | **Total** | Available data | | **Male** | Available data | **<1 yr** | **1-5 yrs** | **6-17 yrs** | **18-65 yrs** | **>65yrs** | Available data | **Rainy** | Available data | **Central Agglomeration** | **West Guiana** | **East Guiana** | **Center region** | **Savannah region** | **Others*** |
| **Protozoaires** | **1225 (56.5)** |  | |  |  |  |  |  |  |  |  |  |  |  |  |  |  |  |  |
| *Entamoebahistolytica/dispar* | 167 (7.7) | 166 | | 99 (59.6) | 167 | 3 (1.8) | 12 (7.2) | 16 (9.6) | 125 (74.8) | 11 (6.6) | 167 | 101 (60.5) | 163 | 93 (57.1) | 48 (29.4) | 21 (12.9) | 0 (0.0) | 0 (0.0) | 1 (0.6) |
| *Entamoeba coli* | 287 (13.3) | 280 | | 174 (62.1) | 287 | 3 (1.0) | 30 (10.5) | 46 (16.0) | 190 (66.2) | 18 (6.3) | 287 | 174 (60.6) | 286 | 130 (45.5) | 94 (32.9) | 61 (21.3) | 0 (0.0) | 0 (0.0) | 1 (0.3) |
| *Cryptosporidium* | 42 (1.9) | 42 | | 23 (54.8) | 42 | 13 (31.0) | 22 (52.4) | 1 (2.4) | 5 (11.9) | 0 (0.0) | 42 | 24(57.1) | 26 | 13(50.0) | 10 (38.5) | 2 (7.7) | 0 (0.0) | 0 (0.0) | 1 (3.8) |
| *Entamoeba hartmanni* | 193 (8.9) | 190 | | 120 (63.2) | 193 | 1 (0.5) | 18 (9.3) | 26 (13.5) | 142 (73.6) | 6 (3.1) | 193 | 125 (64.8) | 189 | 113 (59.8) | 49 (25.9) | 26 (13.7) | 1 (0.6) | 0 (0.0) | 0 (0.0) |
| *Endolimax nana* | 93 (4.3) | 92 | | 50 (54.3) | 93 | 0 (0.0) | 10 (10.8) | 12 (12.9) | 68 (73.1) | 3 (3.2) | 93 | 56 (60.2) | 93 | 41 (44.1) | 43 (46.2) | 7 (7.5) | 0 (0.0) | 0 (0.0) | 2(2.2) |
| *Pseudolimax butschilii.* | 9 (0.4) | 9 | | 4 (44.4) | 9 | 0 (0.0) | 2 (22.2) | 0 (0.0) | 7 (77.8) | 0 (0.0) | 9 | 6 (66.7) | 9 | 4 (44.4) | 2 (22.3) | 3 (33.3) | 0 (0.0) | 0 (0.0) | 0 (0.0) |
| *Isospora belli* | 7 (0.3) | 7 | | 2 (28.6) | 7 | 0 (0.0) | 1 (14.3) | 0 (0.0) | 5 (71.4) | 1 (14.3) | 7 | 3 (42.9) | 7 | 2 (28.6) | 5 (71.4) | 0 (0.0) | 0 (0.0) | 0 (0.0) | 0 (0.0) |
| *Giardia intestinalis* | 233 (10.8) | 229 | 140 (61.1) | | 232 | 20 (8.6) | 118 (50.9) | 17 (7.3) | 73 (31.5) | 4 (1.7) | 233 | 151 (64.8) | 229 | 61 (26.6) | 128 (55.9) | 40 (17.5) | 0 (0.0) | 0 (0.0) | 0 (0.0) |
| *Trichomonas intestinalis* | 6 (0.3) | 6 | 4 (66.7) | | 6 | 0 (0.0) | 2 (33.3) | 1 (16.7) | 3 (33.3) | 0 (0.0) | 6 | 3 (50.0) | 6 | 0 (0.0) | 6 (100.0) | 0 (0.0) | 0 (0.0) | 0 (0.0) | 0 (0.0) |
| *Chilomastix mesnili* | 34 (1.6) | 34 | 17 (50.0) | | 34 | 0 (0.0) | 9 (26.5) | 5 (14.7) | 16 (47.1) | 4 (44.4) | 34 | 20 (58.8) | 33 | 6 (18.2) | 22 (66.7) | 4 (12.1) | 0 (0.0) | 0 (0.0) | 1 (3.0) |
| *Retortamonas intestinalis* | 7 (0.3) | 7 | 5 (71.4) | | 7 | 0 (0.0) | 0 (0.0) | 1 (14.3) | 6 (85.7) | 0 (0.0) | 7 | 4 (57.1) | 7 | 2 (28.6) | 4 (57.1) | 1 (14.3) | 0 (0.0) | 0 (0.0) | 0 (0.0) |
| *Enteromonas hominis* | 8 (0.4) | 7 | 6 (85.7) | | 8 | 1 (0.1) | 2 (25.0) | 2 (25.0) | 2 (25.0) | 1 (12.5) | 8 | 6 (75.0) | 8 | 2 (25.0) | 5 (62.5) | 1 (12.5) | 0 (0.0) | 0 (0.0) | 0 (0.0) |
| *Blastocystis hominis* | 139 (6.4) | 137 | 79 (57.7) | | 139 | 2 (1.4) | 12 (8.6) | 18 (12.9) | 96 (69.1) | 11 (7.9) | 139 | 57 (41.0) | 139 | 84 (60.4) | 37 (26.6) | 17 (12.2) | 0 (0.0) | 0 (0.0) | 1 (0.8) |
| **Helminthes** | **941 (43.5)** |  |  | |  |  |  |  |  |  |  |  |  |  |  |  |  |  |  |
| *Ancylostoma sp* | 546 (25.2) | 542 | 426 (78.6) | | 544 | 6 (1.1) | 17 (3.1) | 35 (6.4) | 471 (86.6) | 15 (2.8) | 546 | 325 (59.5) | 536 | 250 (46.6) | 192 (35.8) | 70 (13.2) | 7 (1.3) | 1 (0.2) | 16 (2.9) |
| *Ascaris lumbricoïdes* | 67 (3.1) | 64 | 39 (60.9) | | 67 | 6 (9.0) | 21 (31.3) | 17 (25.4) | 18 (26.9) | 5 (7.5) | 67 | 35 (52.2) | 67 | 30 (44.8) | 20 (29.8) | 17 (25.4) | 0 (0.0) | 0 (0.0) | 0 (0.0) |
| *Enterobius vermicularis* | 15 (0.7) | 14 | 9 (64.3) | | 15 | 0 (0.0) | 2 (13.3) | 4 (26.7) | 8 (53.3) | 1 (6.7) | 15 | 9 (60.0) | 15 | 8 (53.3) | 3 (20.0) | 4 (26.7) | 0 (0.0) | 0 (0.0) | 0 (0.0) |
| *Strongyloides stercoralis* | 235 (10.9) | 233 | 189 (81.1) | | 235 | 4 (1.7) | 8 (3.4) | 9 (3.8) | 180 (76.6) | 34 (14.5) | 235 | 152 (64.7) | 232 | 132 (56.9) | 69 (29.7) | 23 (10.0) | 1 (0.4) | 6 (2.6) | 1 (0.4) |
| *Trichuris trichiura* | 45 (2.1) | 45 | 25 (55.6) | | 45 | 1 (2.2) | 5 (11.1) | 5 (11.1) | 30 (66.6) | 4 (8.9) | 45 | 26 (57.8) | 43 | 32 (74.4) | 10 (23.3) | 1 (2.3) | 0 (0.0) | 0 (0.0) | 0 (0.0) |
| *Capillaria sp* | 4 (0.2) | 4 | 1 (25.0) | | 4 | 1 (25.0) | 2 (50.0) | 0 (0.0) | 1 (25.0) | 0 (0.0) | 4 | 3 (75.0) | 4 | 3 (75.0) | 1 (25.0) | 0 (0.0) | 0 (0.0) | 0 (0.0) | 0 (0.0) |
| *Hymenolepis nana* | 24 (1.1) | 19 | 13 (68.4) | | 24 | 1 (4.2) | 7 (29.2) | 6 (25.0) | 9 (37.5) | 1 (4.2) | 24 | 12 (50.0) | 22 | 3 (13.65) | 16 (72.7) | 3 (13.65) | 0 (0.0) | 0 (0.0) | 0 (0.0) |
| *Hymenolepis diminuta* | 3 (0.2) | 3 | 1 (33.3) | | 3 | 1 (33.3) | 2 (66.7) | 0 (0.0) | 0 (0.0) | 0 (0.0) | 3 | 1 (33.3) | 3 | 1 (33.3) | 1 (33.3) | 1 (33.3) | 0 (0.0) | 0 (0.0) | 0 (0.0) |
| *Moat* | 2 (0.2) | 2 | 1 (50.0) | | 2 | 0 (0.0) | 0 (0.0) | 0 (0.0) | 1 (50.0) | 1 (50.0) | 2 | 1 (50.0) | 2 | 0 (0.0) | 1 (50.0) | 1 (50.0) | 0 (0.0) | 0 (0.0) | 0 (0.0) |

*France Metropolis, Surinam, Brasil, French West Indies and Africa.
