# Supplementary material for: Prevalence of intestinal parasite among patients attending two hospitals in French Guiana: A 6-year retrospective study
Source: PLoS Negl Trop Dis. 2021 Feb 5;15(2):e0009087. doi: 10.1371/journal.pntd.0009087 (PMC7891781; doi:10.1371/journal.pntd.0009087)
Supplement: S3 Table — (DOCX) [file pntd.0009087.s004.docx]

**S3 Table. The most common combinations of parasites**

|  | **Available data** | **Number of patients** |
| --- | --- | --- |
| **Combination of 2 parasites** (n, %) |  |  |
| *Ancylostoma+ Giardia intestinalis* | 471 | 41 (8.7) |
| *Ancylostoma + Strongyloides stercoralis* | 471 | 34 (7.2) |
| *Ancylostoma+ Entamoeba coli* | 471 | 25 (5.3) |
| *Entamoeba coli + Entamoeba hartmanni* | 471 | 20 (4.2) |
| *Entamoeba coli +Entamoeba histolytica/dispar* | 471 | 16 (3.4) |
| *Ancylostama + Entamoeba hartmanni* | 471 | 14 (3.0) |
| *Entamoeba hartmanni +Endolimax nana* | 471 | 14 (3.0) |
| *Ancylostoma + Blastocystis hominis* | 471 | 11 (2.3) |
| *Ancylostoma + Trichuris trichiura* | 471 | 10 (2.1) |
| *Entamoeba histolytica/dispar + Entamoeba hartmanni* | 471 | 10 (2.1) |
| *Ancylostoma + Entamoeba histolytica/dispar* | 471 | 8 (1.7) |
| *Strongyloides stercoralis + Blastocystis hominis* | 471 | 8 (1.7) |
| *Strongyloides stercoralis + Giardia intestinalis* | 471 | 7 (1.5) |
| *Entamoeba coli+ Giardia intestinalis* | 471 | 6 (1.3) |
| *Giardia intestinalis + Ascaris lumbricoides* | 471 | 6 (1.1) |
| *Entamoeba coli + Blastocystis hominis* | 471 | 5 (1.1) |
| **Combination of 3 parasites** (n, %) |  |  |
| *Ancylostoma + Entamoeba coli + Entamoeba hartmanni* | 471 | 8 (1.7) |
| *Entamoeba coli + Entamoeba histolytica/dispar + Entamoeba hartmanni* | 471 | 8 (1.7) |
| *Ancylostama + Strongyloides stercoralis + Giardia intestinalis* | 471 | 5 (1.1) |
