# Supplementary material for: Prevalence of intestinal parasite among patients attending two hospitals in French Guiana: A 6-year retrospective study
Source: PLoS Negl Trop Dis. 2021 Feb 5;15(2):e0009087. doi: 10.1371/journal.pntd.0009087 (PMC7891781; doi:10.1371/journal.pntd.0009087)
Supplement: S4 Table — (DOCX) [file pntd.0009087.s005.docx]

**S4 Table. Characteristics of patients with mixed infections**

|  | **Available data** | **Poly parasitic patients**  **n=** 471 | **Available data** | **Mono parasitic patients**  **n=** 1,050 | **p** |
| --- | --- | --- | --- | --- | --- |
| **Male gender**(n, %) | 462 | 309 (66.9) | 1,037 | 685 (66.0) | 0.76 |
|  |  |  |  |  |  |
| **Median age** (years) (n, %) | 470 | 30.0 years | 1,050 | 34.5 years |  |
| < 1 year |  | 14 (3.0) |  | 35 (3.3) | 0.88 |
| 1-5 years |  | 71 (15.1) |  | 146 (13.9) | 0.32 |
| 6-17 years |  | 60 (12.8) |  | 73 (7.0) | **<0.001** |
| < 18 years |  | 145 (30.9) |  | 255 (24.3) | **<0.001** |
| 18-64 years |  | 300 (64.8) |  | 729 (69.4) | 0.10 |
| >=65 years |  | 25 (5.3) |  | 66 (6.3) | 0.56 |
|  |  |  |  |  |  |
| **Years of diagnosis** (n, %) | 471 |  | 1,050 |  |  |
| 2011 |  | 92 (19.5) |  | 194 (18.5) | 0.62 |
| 2012 |  | 85 (18.04) |  | 170 (16.2) | 0.37 |
| 2013 |  | 66 (14.0) |  | 135 (12.9) | 0.57 |
| 2014 |  | 76 (16.1) |  | 173 (16.5) | 0.94 |
| 2015 |  | 109 (23.1) |  | 187 (17.8) | **0.02** |
| 2016 |  | 43 (9.1) |  | 191 (18.2) | **<0.001** |
|  |  |  |  |  |  |
| **Rainy Season** (n, %) | 471 | 240 (51.0) | 1,050 | 491 (46.8) | 0.09 |
|  |  |  |  |  |  |
| **Linving places** (n, %) | 464 |  | 1,021 |  |  |
| Central Agglomeration |  | 208 (44.8) |  | 525 (51.4) | 0.87 |
| West Guiana |  | 181 (39.0) |  | 375 (36.7) | 0.42 |
| East Guiana |  | 69 (14.9) |  | 89 (8.7) | **<0.001** |
| Center region |  | 1 (0.2) |  | 7 (0.7) | 0.45 |
| Savannah region |  | 0 (0.0) |  | 7 (0.7) | 0.11 |
| Others* |  | 5 (1.1) |  | 18 (1.8) | 0.37 |

*France Metropolis, Surinam, Brasil, French West Indies, Africa
